# Sequence Matching between Hemagglutinin and Neuraminidase through Sequence Analysis Using Machine Learning

**DOI:** 10.3390/v14030469

**Published:** 2022-02-23

**Authors:** He Wang, Yongjian Zang, Yizhen Zhao, Dongxiao Hao, Ying Kang, Jianwen Zhang, Zichen Zhang, Lei Zhang, Zhiwei Yang, Shengli Zhang

**Affiliations:** MOE Key Laboratory for Nonequilibrium Synthesis and Modulation of Condensed Matter, School of Physics, Xi’an Jiaotong University, Xi’an 710049, China; w1047181605@stu.xjtu.edu.cn (H.W.); zyj198984@stu.xjtu.edu.cn (Y.Z.); zyz9856@stu.xjtu.edu.cn (Y.Z.); yuzhouweijia@stu.xjtu.edu.cn (D.H.); kangying0048@stu.xjtu.edu.cn (Y.K.); cheung1998@stu.xjtu.edu.cn (J.Z.); ozhangzc@stu.xjtu.edu.cn (Z.Z.); zhangleio@xjtu.edu.cn (L.Z.)

**Keywords:** influenza A viruses, hemagglutinin, neuraminidase, viral evolution, sequence analysis, machine learning

## Abstract

To date, many experiments have revealed that the functional balance between hemagglutinin (HA) and neuraminidase (NA) plays a crucial role in viral mobility, production, and transmission. However, whether and how HA and NA maintain balance at the sequence level needs further investigation. Here, we applied principal component analysis and hierarchical clustering analysis on thousands of HA and NA sequences of A/H1N1 and A/H3N2. We discovered significant coevolution between HA and NA at the sequence level, which is closely related to the type of host species and virus epidemic years. Furthermore, we propose a sequence-to-sequence transformer model (S2STM), which mainly consists of an encoder and a decoder that adopts a multi-head attention mechanism for establishing the mapping relationship between HA and NA sequences. The training results reveal that the S2STM can effectively realize the “translation” from HA to NA or vice versa, thereby building a relationship network between them. Our work combines unsupervised and supervised machine learning methods to identify the sequence matching between HA and NA, which will advance our understanding of IAVs’ evolution and also provide a novel idea for sequence analysis methods.

## 1. Introduction

Against the background of coronavirus disease 2019 [1], the influenza A viruses (IAVs) [2] continue to pose a risk and endanger human health. In long-term research, it has been found that two major surface glycoproteins, i.e., hemagglutinin (HA) [3] and neuraminidase (NA) [4], are involved in the process of virus infectivity, replication, and transmission [5]. To date, 18 HA and 11 NA subtypes have been identified, and over 120 combinations have been documented in nature [6]. The antigenic drift and reassortment of HA and NA cause a series of pathogenic and epidemic strains [7,8,9]; among them, the A/H1N1 and A/H3N2 subtypes circulate in the human population and give rise to seasonal outbreaks [10].

HA and NA are a pair of functional antagonist proteins: HA binds to sialic acid through its receptor binding site, while NA is a receptor-destroying enzyme that cleaves α2-3- and α2-6-linked sialic acids [11]. The functional balance between HA and NA is necessary for viral production and interspecies transmission [12]. Viral particles need to penetrate a gel-like mobile mucus layer under the co-regulation of HA and NA in order to reach and, subsequently, infect the underlying epithelial cells [13,14]. The HA–NA–receptor balance promotes the efficient absorption and separation of the virus from host cells [15]. The level of co-existing HA can impact NA enzymatic activities; in addition, NA function against different substrates is correlated with the HA receptor specificity [16]. To compensate for the ability of NA to remove sialic acid residues from the virion surface, some gene mutations associated with a decreasing affinity will occur in HA [17]. Nevertheless, the way in which HA and NA maintain balance at the sequence level remains unclear.

A large amount of influenza virus sequence information is provided by laboratories around the world, contributing to basic databases for virus sequence analysis, such as virus evolution [18,19]. Bao et al. used an integrated clustering model to analyze the distribution and evolution of A/H1N1 HA segments before 2018 and discovered that, every year, there was a dominant strain type [20]. Yin et al. proposed a time-series mutation prediction model based on attention-based recurrent neural networks to predict next-generation HA sequences according to the existing HA sequences [21]. Ward et al. investigated how the association with different NA subtypes (N1, N2, N3, and N7) influences the evolution of H7 using a Bayesian stochastic mutational mapping approach [22]. Various machine learning methods have advanced the research of virus evolution and have paved the way for us to study the inherent balance of HA and NA.

In this paper, we study the sequence matching between HA and NA in A/H1N1 and A/H3N2 strains based on sequence analysis. Principal component analysis and hierarchical cluster analysis are employed to explore the distribution and evolution of HA and NA, revealing a close match between them. We then propose an attention-based neural network model, named the sequence-to-sequence transformer model (S2STM), to map relationships between HA and NA sequences. The S2STM has good effectiveness, robustness, and realizes mutual mapping between HA and NA sequences. Our work combines unsupervised and supervised machine learning methods to identify the HA–NA–receptor balance at the sequence level; this will advance our understanding of IAV evolution and provide novel insights into the coevolution between HA and NA, which will also promote the sequence analysis methods.

## 2. Materials and Methods

### 2.1. Data Collection and Preparation

All “full-length plus” sequences of HA and NA in A/H1N1 and A/H3N2 were downloaded from the NCBI Influenza Virus Resource [23] (https://www.ncbi.nlm.nih.gov/genomes/FLU/Database/nph-select.cgi?go=database accessed on 17 February 2022) until July 20, 2020. Sequence alignments were performed using MEGA X [24] v10.2.6. We deleted the insertions and used “–” to fill in the missing data, so as to ensure that every HA and NA had a sequence length of 566 and 469, respectively. HA and NA were matched according to whether they corresponded to the same strain name; if multiple strains had the same HA and NA at the same time, we retained only one strain randomly. Finally, 11,464 A/H1N1 and 11,677 A/H3N2 strains were retained as the primitive data.

### 2.2. Principal Component Analysis and Hierarchical Cluster Analysis

As shown in Figure S1, we established a 2D matrix of $\left(m,\;l\right)$ dimensionality for each sequence, where m is the number of amino acid markers and l is the sequence length. For sequence s , if s has an amino acid “A” at site j whose index is i , then $M_{ij}=1$ ; otherwise, $M_{ij}=0$ . M is then smoothed into a 1D matrix with length m×l , and, thereby, all flattened sequences form a larger 2D matrix of dimensions ( n, m×l ), where n is the number of sequences. Using principal component analysis (PCA) [25], we reduced it to a matrix with dimensions ( n,k ), where k is the number of reserved dimensions. Here, the PCA was performed using the “Sklearn” [26,27] Python library, while the hierarchical clustering [28] was operated using the “Scipy” [29] Python library.

### 2.3. Sequence-to-Sequence Transformer Model

Vaswani et al. developed a simple network model named “Transformer” [30] based solely on attention mechanisms, instead of recurrence and convolutions. This general architecture can learn long-range dependencies and attend to different positions of the input sentence to compute its representation. The attention weights behind the transformer provide insight into the complex internal relationship between sentences (e.g., grammar). Analogously, we constructed the S2STM based on the multi-head attention mechanism to study the mapping relationship between the HA and NA sequences.

#### 2.3.1. Word Set and Data Division

To prepare the training data to be suitable for the S2STM, primitive sequences were decomposed into shifted overlapping residues in a window of 3. Each HA sequence was depicted as a list of 566 3-g, and each NA sequence was described as a list of 469 3-g. For A/H1N1, we obtained a HA word set with a size of 5929 and an NA word set with a size of 5034; for A/H3N2, a HA word set of 5580 words and an NA word set of 5036 words were acquired. These words were converted into numerical representations used as indices into an embedding.

The entire training and testing processes were performed using TensorFlow [31] v2.0.4. We divided the prepared data in different ways as needed: (1) to verify the robustness of the model, the data were randomly divided into training and testing samples with a ratio of 0.8:0.2, termed the “R” method; (2) to test the translation ability for the latest HA–NA pairs, the strains before 2019 (2019 was not included) were used for training, and the remaining data for testing, termed the “T” method; (3) to observe the overall translation ability of the locally trained model, partial “Human” clusters were extracted, of which strains were divided into training and testing sets with a ratio of 0.8:0.2, termed the “H*” method.

#### 2.3.2. Multi-Head Attention Mechanism

The self-attention mechanism can be described as mapping a query and a set of key–value pairs to an output. It takes Q (query), K (key), and V (value) with dimensions $d_{k}$ , $d_{k}$ , and $\;d_{v}$ , respectively, as inputs, and the equation used to calculate the output weights is [30]

(1) $$\mathrm{Attention}\left(Q,\;K,\;V\right)=\mathrm{softmax}\left(\frac{{\mathrm{QK}}^{T}}{\sqrt{d_{k}}}\right)V$$

Developed from self-attention, multi-head attention (MHA) allows the model to jointly attend to information from different representation subspaces at different positions. The MHA with n heads can be calculated as follows [30]:

(2) $$MultiHead\left(Q,\;K,\;V\right)=Contact\left(head_{1},\;\ldots ,\;head_{n}\right)W^{O}$$

(3) $$\mathrm{where}\;head_{i}=Attention\left(QW_{i}^{Q},\;KW_{i}^{K},\;VW_{i}^{V}\right)$$

#### 2.3.3. Base Model

**Figure 1:**
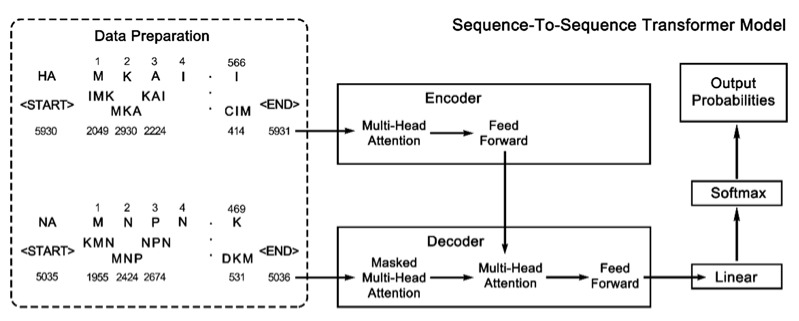
Process of sequence-to-sequence transformer model (S2STM). The hemagglutinin (HA) and neuraminidase (NA) sequences are discretized into “sentences” composed of triplets and then numbered, which are used as indices into an embedding. A start sign “<START>” and an end sign “<END>” are added at both ends of the sentence. S2STM is mainly composed of an encoder, a decoder, and a final linear layer, where a multi-head attention mechanism is applied.

The S2STM consists of an encoder, a decoder, and a final linear layer, with the complete workflow shown in Figure 1. The encoder and decoder are composed of n_layers=4 identical layers. Each encoder layer has two sub-layers, including a multi-head attention mechanism and a position-wise fully connected feed-forward layer. Each decoder layer consists of three sub-layers, including a masked multi-head attention mechanism, a multi-head attention mechanism, and a position-wise fully connected feed-forward layer. The dimensionality of input and output is $d_{model}=128$ ; the number of parallel attention layers, or heads, is set as n_heads=8 ; the inner layer has a dimensionality of $d_{ff}=512$ ; and a dropout rate of $P_{drop}=0.1$ is applied. The output of the decoder is the input to the linear layer, and its output is returned. We used the Adam optimizer with $\beta_{1}=0.9$ , $\;\beta_{2}=0.98$ , and $\varepsilon =10^{-9}$ .

## 3. Results

### 3.1. Hierarchical Clustering Analysis

**Figure 2:**
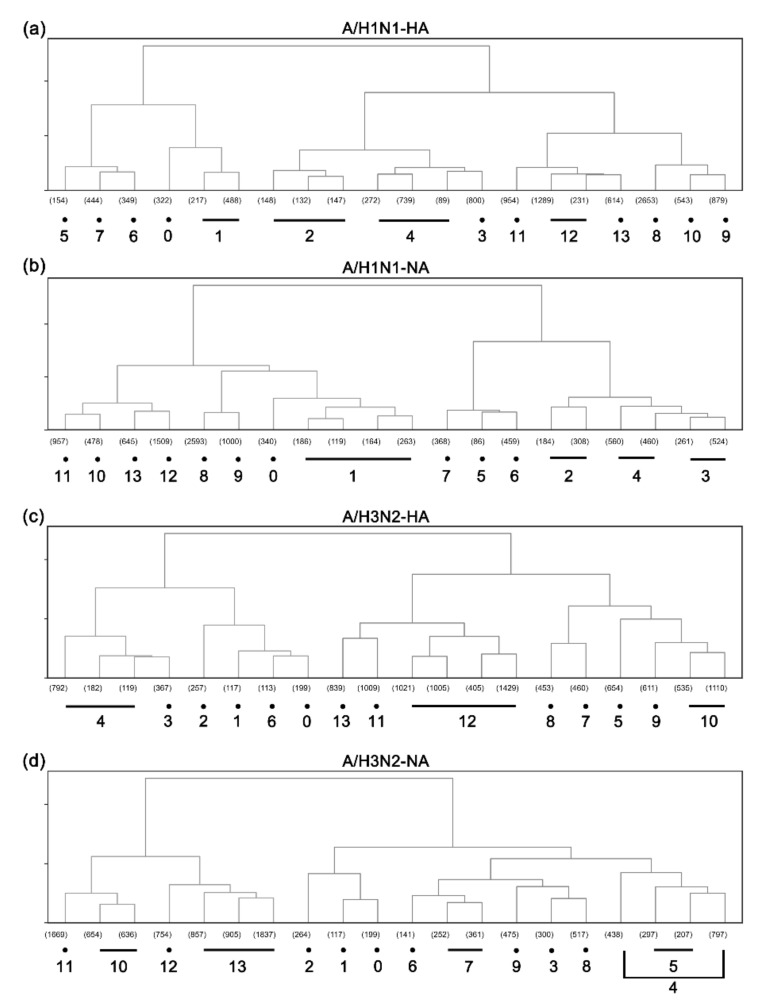
Hierarchical clustering analysis results. (**a**) A/H1N1-HA; (**b**) A/H1N1-NA; (**c**) A/H3N2-HA; (**d**) A/H3N2-NA. The number of virus strains in each initial cluster is indicated in brackets. Clusters are merged and reordered manually (as indicated at the bottom).

We performed hierarchical clustering analysis on the PCA matrices of HA and NA with reduced dimensionality, and, interestingly, these clusters were closely related to the host species and epidemic years of the viruses. Then, we manually merged the clusters according to their similarity and whether there were similar host species and time distribution range. Finally, we retained 14 clusters of HA and NA for A/H1N1 and 14 clusters for A/H3N2 (Figure 2). Almost every cluster has a dominant host species, strains with which account for more than 80% of each cluster. The time distribution range of most clusters is similar to a normal distribution, going through the beginning, bursting, and falling or disappearing.

**Figure 3:**
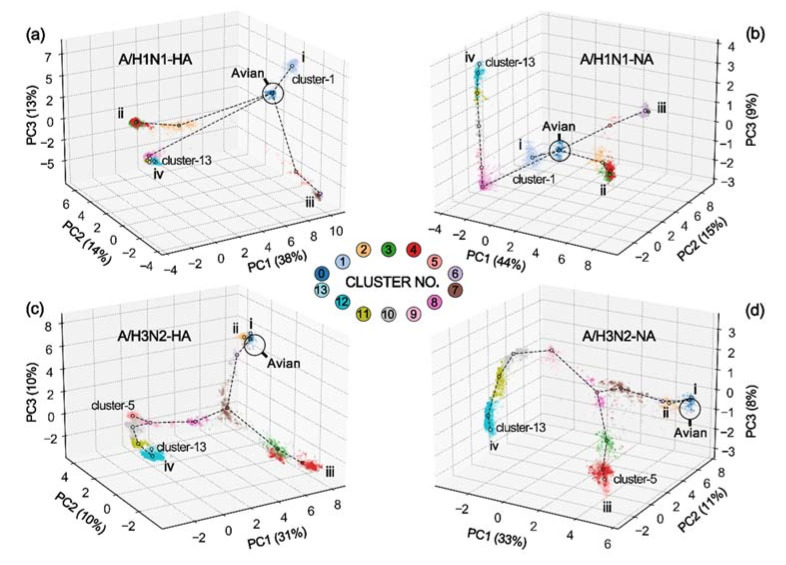
Hierarchical clustering analysis based on principal component analysis (PCA) matrices with dimensionality reduction. (**a**) A/H1N1-HA; (**b**) A/H1N1-NA; (**c**) A/H3N2-HA; (**d**) A/H3N2-NA. Each subset is divided into 14 clusters (indicated in Figure 2), which are grouped into different evolution branches (i, ii, iii, and iv) starting from the “Avian” cluster. The X-axis, Y-axis, and Z-axis represent the projections to the first three principal components (PCs): PC1, PC2, and PC3, respectively.

The first three principal components (PCs) retained over 50% of the information, showing different temporal evolutions in A/H1N1 and A/H3N2 (Figure 3). In A/H1N1, we summarize four evolutionary branches starting from the “Avian” cluster (cluster-0): branch-i is composed of cluster-1 that takes “Swine” as the main host, which is mainly distributed from 2003 to 2017; branch-ii including cluster-2–4 reflects another evolution of “Swine” that has been active until the data collection time; branch-iii consists of cluster-5–7, which is dominated by “Human” before 2010; and branch-iv represents another evolution of “Human”, consisting of cluster-8–13, that starts from 2009 and continues to the data collection time. There were some differences between the HA and NA groups. From the 3D map, branch-iv of HA is close to cluster-2 located in branch-iii (Figure 3a), while, in NA, it is near cluster-1, located in branch-i (Figure 3b). In comparison to HA, branch-iv of NA shows a linear evolution, having a long evolutionary distance on PC3.

In contrast to A/H1N1, A/H3N2 has a more obvious mainline (branch-iv), which is composed of cluster-0 (“Avian”-dominated) and cluster-6–13 (“Human”-dominated). It derived various branches in different periods: cluster-0 derived cluster-1 (branch-i, “Canine”-dominated) and cluster-2 (branch-ii, “Swine”-dominated) in the early days; in approximately 2003, cluster-7 of HA derived cluster-3–5 (branch-iii, “Swine”-dominated), and cluster-8 of NA derived cluster-3–4 (branch-iii, “Swine”-dominated). Differently, cluster-5 (“Swine” dominated) of HA is adjacent to branch-iv (Figure 3c), while that of NA is located in branch-iii (Figure 3d). In general, the A/H3N2 clusters have good continuity, whereas there are many “spans” in A/H1N1. We then define the branch evolution distance as follows:

(4) $$d_{b}=\sum \;\frac{\Delta S_{i,i+1}}{r_{Avian}}\;$$

where $d_{b}$ is the evolutionary distance of one branch, $r_{Avian}$ is the radius of the “Avian” cluster, and ${\Delta S}_{i,\;i+1}$ is the distance between clusters i and i+1 located in this branch. Every branch starts with the “Avian” cluster. As shown in Table 1, the evolutionary distance of HA’s branch is much longer than that of the matching NA’s branch.

### 3.2. Coevolution between HA and NA

**Figure 4:**
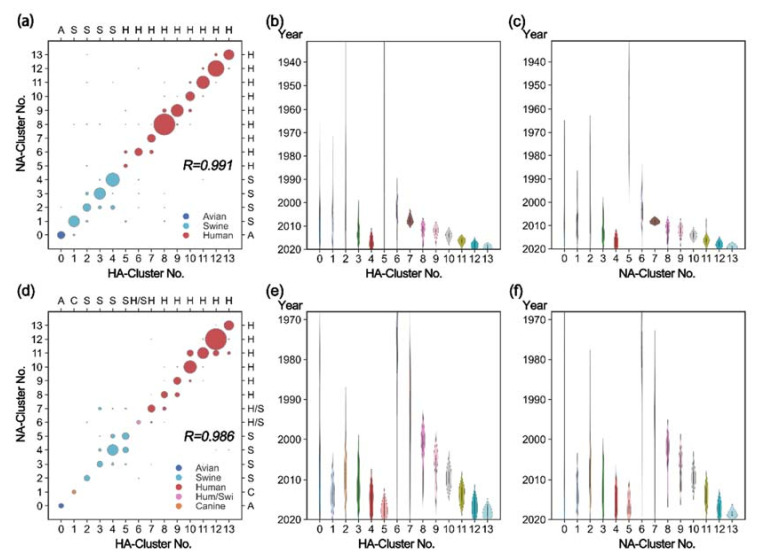
Cluster matching between HA and NA clusters. (**a**) Correlation between HA and NA clusters in A/H1N1. The color of the circle represents the type of host species and the size represents the number of contained strains. The more points on the diagonal, the stronger the linear correlation is; this is measured using Pearson coefficient with R=0.991 in A/H1N1. (**b**) The violin plot shows the time evolution of each HA cluster of A/H1N1. (**c**) Time evolution of NA clusters of A/H1N1. (**d**) Correlation between HA and NA clusters in A/H3N2 with R=0.986 . (**e**) Time evolution of HA clusters of A/H3N2. (**f**) Time evolution of NA clusters of A/H3N2. The HA-cluster number and NA-cluster number are indicated in Figure 2. The abbreviations “A” (“Avian”), “C” (“Canine”), “S” (“Swine”), and “H” (“Human”) represent the dominant host species in each cluster. “H/S” (“Human/Swine”) indicates that both host species account for a large proportion.

The 3D projections of HA and NA on PC1, PC2, and PC3 have topological similarities in A/H1N1 and A/H3N2, suggesting a correlation between HA and NA. After analyzing the composition of virus strains (Figure 4), we found that, in general, HA proteins in one HA cluster mainly matched NA proteins in one specific NA cluster to form strains. To quantitatively describe the matching degree of HA and NA clusters, the Pearson coefficient was calculated as follows:

(5) $$\rho_{X,Y}=\frac{E\left[\left(X-\mu_{X}\right)\left(Y-\mu_{Y}\right)\right]}{\sigma_{X}\sigma_{Y}}$$

where X and Y are lists of the cluster tags of HA and NA (indicated in Figure 2), respectively. The closer the value is to 1, the stronger the linear relationship will be. The correlation coefficient of A/H1N1 was 0.991, and that of A/H3N2 was 0.986, implying a strong linear relationship between HA and NA. The paired HA and NA clusters generally have similar host species as well as epidemic years (Figure 4), suggesting that the sequence matching between HA and NA is host-dependent and time-related. In particular, the clusters in each branch show a progressive relationship over time, indicating that the matching may evolve over time as well.

Although the relationship between HA and NA is rapid and intense, its mapping is not based on simple sequence similarity. In other words, some HA clusters with similar sequences may match NA clusters with large sequence differences. For example, as shown in Figure 3, some HA clusters close to “Avian” (cluster-0) may prefer NA clusters far away from “Avian”. In addition, it is noteworthy that a small amount of HA and NA will break through the cluster limitation for cross-combinations, especially those located in different branches or belonging to different host species. This phenomenon may be related to viral rearrangement or recombination.

### 3.3. Translation Performance of S2STM

The S2STM was trained and tested from HA to NA (“HA-to-NA”) or NA to HA (“NA-to-HA”) (Figures S2–S5). Taking A/Wyoming/07/2013(H1N1) as an example (Figures S6 and S7), we respectively translated its HA and NA sequence using S2STM, and achieved good results. The attention maps in Figures S6b and S7b give the attention weights in the translation process, which visualize the internal working of the model. In general, as listed in Table 2, all of the training results achieved testing accuracies larger than 0.99, suggesting that the S2STM has good robustness; it can successfully learn and establish the mapping relationship between HA and NA sequences. The model also provided good accuracy when we trained and tested it using the strains before and after 2019, showing the ability to translate future HA–NA pairs. Higher accuracy could be obtained when we only used the dataset composed of “Human” strains after 2009. These performances prove the possibility of mastering the balance laws between the HA and NA with the help of machine learning.

**Figure 5:**
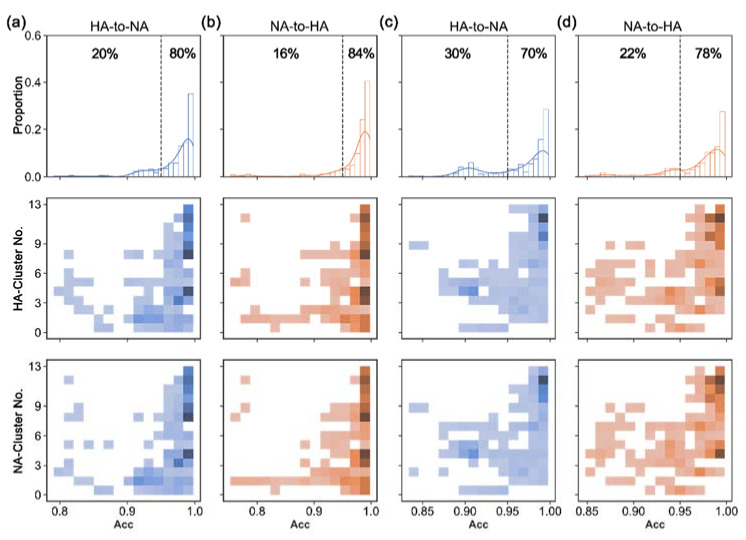
Distribution of translation accuracies using selected strains in the testing dataset. (**a**) A/H1N1, from HA to NA (“HA-to-NA”); (**b**) A/H1N1, from NA to HA (“NA-to-HA”); (**c**) A/H3N2, from HA to NA; (**d**) A/H3N2, from NA to HA. The X-axis represents the translation accuracies; Y-axis respectively indicates the proportion of accuracies, HA-cluster number, and NA-cluster number, from top to bottom. The proportions of strains with translation accuracies greater than 0.95 (or less than 0.95) are indicated in the first line of images. The accuracy distribution of each HA and NA cluster is counted in the second and the third lines of images: the darker the color, the more strains there will be.

To analyze the translation accuracies of S2STM in detail, we chose the virus strains whose HA and NA never appeared in the training set for testing. The trained S2STM (e.g., “R4” in Table 2) was used for sequence translation, with 718 A/H1N1 and 663 A/H3N2 strains being selected. The similarity between the translated and target sequence was computed according to whether the amino acid at each site was the same. We counted the proportion of sequences with translation accuracies larger than 0.95 (Figure 5), which was 80% for “HA-to-NA” and 84% for “NA-to-HA” in A/H1N1, and 70% and 78% in A/H3N2, respectively. Among them, the translation accuracies for HA–NA pairs in cluster-8–13 were generally greater than 0.95.

To estimate the overall translation accuracies of the locally trained model, we trained the S2STM using strains in cluster-8–l2 located in branch-iv (“H*” in Table 2), and randomly selected up to 100 strains from other clusters for translation. According to Table 3, this locally trained model acquired different accuracies on different clusters. Interestingly, the closer the cluster to branch-iv (Figure 3), the higher the accuracy will be. For example, cluster-1 of A/H1N1-NA has accuracy greater than 0.90, whereas that of A/H1N1-HA is only 0.78, where the latter is far away from branch-iv. Cluster-13 is directly located downstream of the training data, and accuracies greater than 0.95 are obtained under the four types of training. In general, the translation ability of the local training model can be compared with the hierarchical clustering analysis results. In summary, the locally trained model is only applicable to target sequences with little difference from the training data.

## 4. Discussion

The balance between HA and NA is important for the infection and transmission of the IAVs [32,33,34]. It does not only exist between different subtypes of HA and NA, but also in the evolution of specific virus subtypes (e.g., A/H1N1 and A/H3N2). Mastering the internal relationship between HA and NA will facilitate research on virus evolution and vaccine or inhibitor design. Although a phylogenetic tree is a good method to study the evolution of protein or gene families, dealing with thousands of sequences will be difficult.

PCA and hierarchical cluster analysis methods were applied to complete the sequence classification, so that we could compare the evolution modes of HA and NA. HA and NA show a one-to-one evolutionary trend to maintain balance. This sequence matching is host-dependent and time-related, and it changes with sequence evolution. When an old strain generates a new independent strain, the old strain will continue to exist and evolve with the new one for some time; however, their evolution directions are different. We conclude that, despite the strains having evolved for many generations, the locally trained model still has a certain ability to translate the old HA–NA pairs. However, when the new strain evolves sufficiently, the old match may not work any longer. Therefore, identifying the direct upstream and downstream of virus strains, and effectively grouping them, will be essential for studying the evolution and balance mechanism.

S2STM could translate the HA sequence into its matching NA, or vice versa. The experimental results show good effectiveness and robustness on both the A/H1N1 and A/H3N2 datasets, especially on the “Human” strains after 2009. The sequence translation between HA and NA paves the road to a clear co-mutation relationship between them, which will guide researchers to avoid the failure of vaccine or inhibitor design caused by the functional balance between HA and NA. There have been many studies on the antigenic variant prediction of HA or NA [35,36,37], where the mapping relationship between HA and NA will improve the prediction accuracy. Therefore, interpreting the attention weights and deriving the exact mapping between HA and NA will have far-reaching research significance.

In this article, we underscore a constructive method combined with some supervised and unsupervised techniques, which will not only advance our understanding of IAV evolution and provide novel insights into the coevolution between HA and NA, but also promote the sequence analysis methods. Our approach has the potential to explore the coevolution between other proteomes or genomes. Using this coevolutionary relationship, we can connect many seemingly unrelated proteomes or genomes, to promote our understanding of the synergistic mechanism between biological macromolecules.

## Figures and Tables

**Table 1:** Branch evolution distances of hemagglutinin (HA) and neuraminidase (NA).

Branch	A/H1N1		A/H3N2	
	HA	NA	HA	NA
i	2.9	1.0	1.1	0.1
ii	10.7	4.5	0.9	0.6
iii	10.0	4.0	14.3	5.7
iv	11.8	6.0	22.5	8.6

**Table 2:** Testing accuracies of S2STM.

Division	A/H1N1		A/H3N2	
	HA-to-NA	NA-to-HA	HA-to-NA	NA-to-HA
R1	0.9930	0.9923	0.9925	0.9927
R2	0.9927	0.9920	0.9919	0.9929
R3	0.9928	0.9918	0.9934	0.9928
R4	0.9925	0.9918	0.9925	0.9926
R5	0.9928	0.9918	0.9931	0.9927
R6	0.9929	0.9921	0.9929	0.9925
R7	0.9934	0.9921	0.9927	0.9927
R8	0.9929	0.9919	0.9931	0.9929
R9	0.9923	0.9918	0.9926	0.9927
R10	0.9930	0.9921	0.9929	0.9929
T	0.9938	0.9931	0.9940	0.9938
H*	0.9954	0.9944	0.9950	0.9949

“R”, “T”, and “H*” are the three ways to divide data (mentioned in the “Section 2”); “HA-to-NA”: translation from HA to NA; “NA-to-HA”: translation from NA to HA.

**Table 3:** Translation accuracies on other clusters of S2STM trained with strains in cluster-8–12.

Cluster	A/H1N1				A/H3N2			
	HA-to-NA		NA-to-HA		HA-to-NA		NA-to-HA	
	Acc	Std	Acc	Std	Acc	Std	Acc	Std
0	0.89	0.008	0.82	0.004	0.85	0.014	0.84	0.009
1	**0.91**	0.010	0.78	0.008	0.83	0.016	0.83	0.006
2	0.83	0.009	**0.90**	0.015	0.85	0.011	0.84	0.006
3	0.83	0.004	**0.92**	0.007	**0.94**	0.021	0.88	0.013
4	0.82	0.003	**0.91**	0.007	**0.93**	0.019	0.87	0.013
5	0.83	0.012	0.80	0.010	**0.90**	0.007	**0.93**	0.010
6	0.82	0.004	0.79	0.003	**0.90**	0.018	0.86	0.018
7	0.81	0.002	0.79	0.003	**0.95**	0.021	**0.90**	0.014
13	**0.98**	0.004	**0.98**	0.011	**0.96**	0.003	**0.96**	0.015

Acc: average accuracies; Std: standard deviation of accuracies. Accuracy greater than 0.90 is bold.

## Data Availability

All data generated or analyzed during this study are included in the main text and Supporting Information, further information and requests may be directed and will be fulfilled by Shengli Zhang (zhangsl@xjtu.edu.cn), the lead contact.

## Supplementary Materials

The following supporting information can be downloaded at: https://www.mdpi.com/1999-4915/14/3/469/s1, Figure S1: Sequence digitization; Figure S2: The training accuracy and loss of sequence-to-sequence transformer model (A/H1N1, from HA to NA); Figure S3: The training accuracy and loss of sequence-to-sequence transformer model (A/H1N1, from NA to HA); Figure S4: The training accuracy and loss of sequence-to-sequence transformer model (A/H3N2, from HA to NA); Figure S5: The training accuracy and loss of sequence-to-sequence transformer model (A/H3N2, from NA to HA); Figure S6: an example for “HA-to-NA” using sequence-to-sequence transformer model; Figure S7: an example for “NA-to-HA” using sequence-to-sequence transformer model.
