# Supplementary material for: Sequence Matching between Hemagglutinin and Neuraminidase through Sequence Analysis Using Machine Learning
Source: Viruses. 2022 Feb 23;14(3):469. doi: 10.3390/v14030469 (PMC8950662; doi:10.3390/v14030469)
Supplement: Supplementary file 1 [file viruses-14-00469-s001.zip › viruses-1581648-supplementary.pdf]

viruses

Supplementary Materials

Sequence Matching between Hemagglutinin and Neuraminidase through Sequence Analysis Using Machine Learning

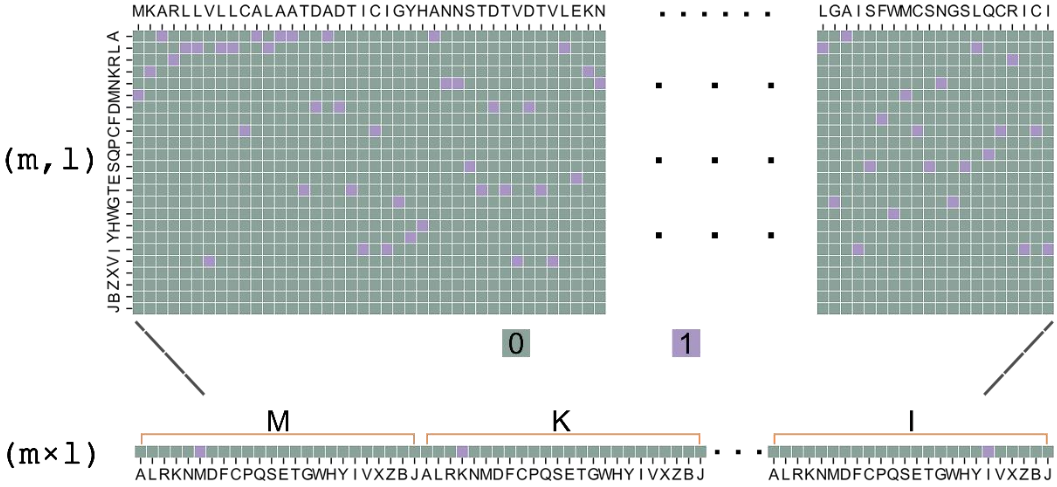

Figure S1. Sequence digitization. One sequence with a length of l residues will be converted to a (m, l) matrix, where m is the number of amino acid abbreviations. This matrix is then flattened into a 1D array with a size of $m \times l$, which is used as the input data for principal component analysis.

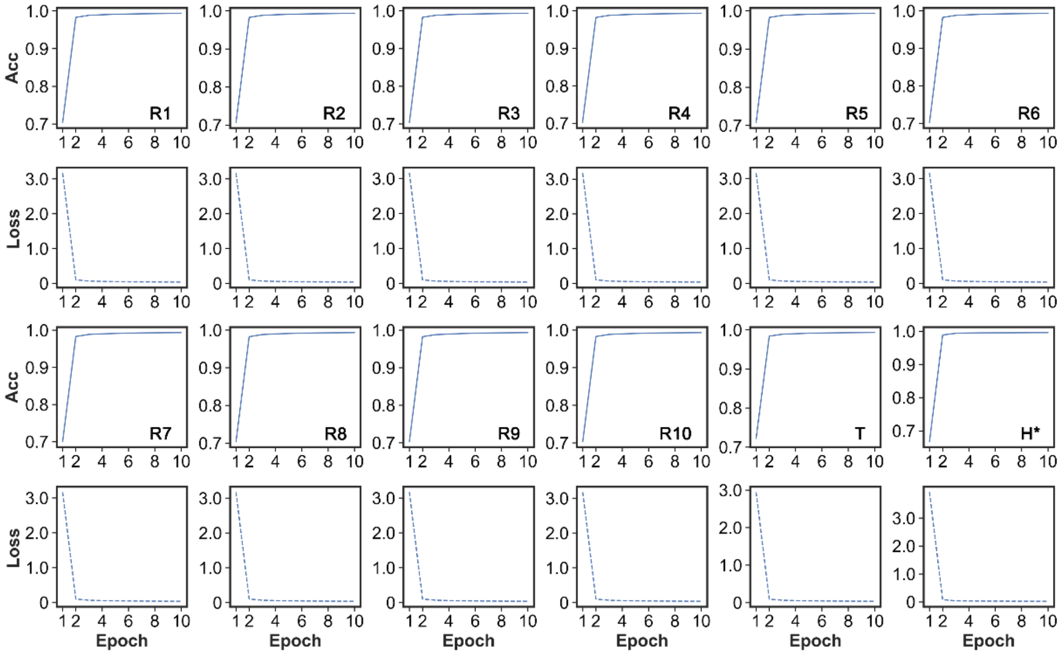

Figure S2. The training accuracy and loss of sequence-to-sequence transformer model (A/H1N1, from HA to NA). As listed in Table 2, "R" 1–10 indicate the randomly divided training data; "T" indicates the division according to time; "H*" indicates the training using "Human" strains after

2009. The X-axis represents the training epoch, where we train the model for a total of 10 epochs. "Acc": the training accuracy; "Loss": the training loss.

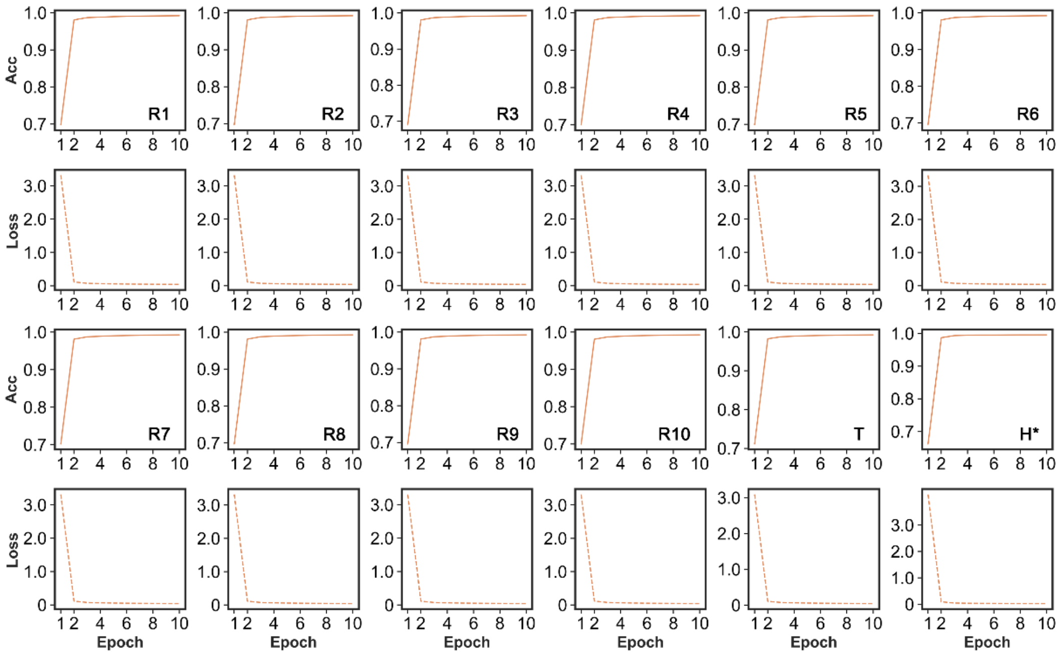

Figure S3. The training accuracy and loss of sequence-to-sequence transformer model (A/H1N1, from NA to HA).

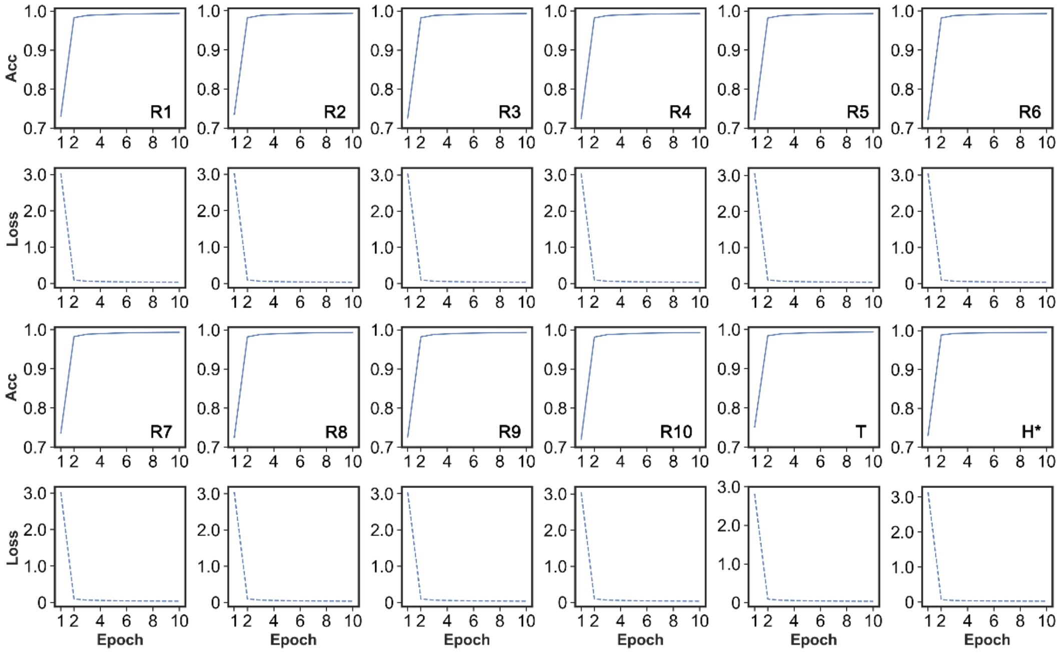

Figure S4. The training accuracy and loss of sequence-to-sequence transformer model (A/H3N2, from HA to NA).

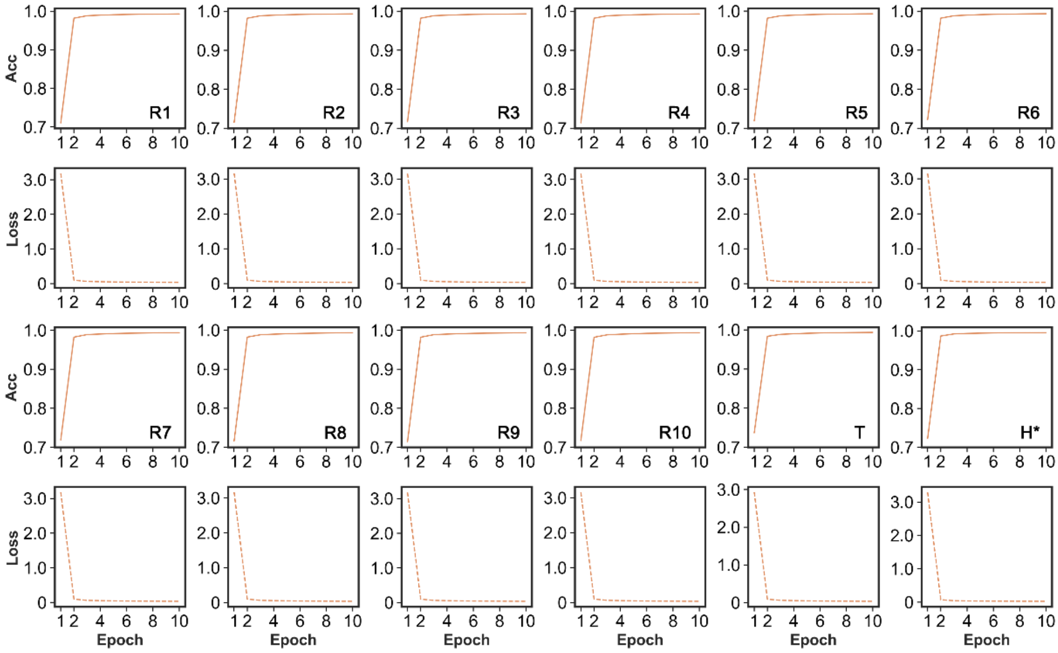

Figure S5. The training accuracy and loss of sequence-to-sequence transformer model (A/H3N2, from NA to HA).

(a)

```
>INPUT_HA

MKATLVVLLY  TFATANADTL  CIGYHANNST  DTVDTVLEKN  VTVTHSVNLL  EDKHNGKLCK  LRGVAPLHLG  KCNIAGWILG  NPECESLSTA
SSWSYIVETS  SSDNGTCYPG  DFINYEELRE  QLSSVSSFER  FEIFPKTSSW  PNHDSNKGVT  AACPHAGAKS  FYKNLIWLVK  KGNSYPKLSQ
SYINDKGKEV  LVLWGIHHPS  TTADQQSLYQ  NADAYVFVGT  SRYSKKFKPE  IAIRPKVRDQ  EGRMNYYWTL  VEPGDKITFE  ATGNLVVPRY
AFTMERNAGS  GIIISDTPVH  DCNTTCQTPE  GAINTSLPFQ  NIHPITIGKC  PKYVKSTKLR  LATGLRNVPS  IQSRGLFGAI  AGFIEGGWTG
MVDGWYGYHH  QNEQGSGYAA  DLKSTQNAID  KITNKVNSVI  EKMNTQFTAV  GKEFNHLEKR  IENLNKKVDD  GFLDIWTYNA  ELLVLLENER
TLDYHDSNVK  NLYEKVRNQL  KNNAKEIGNG  CFEFYHKCDN  TCMESVKNGT  YDYPKYSEEA  KLNREKIDGV  KLESTRIYQI  LAIYSTVASS
LVLVVSLGAI  SFWMCSNGSL  QCRICI

>TARGET_NA

MNPNQKIITI  GSVCMTIGMA  NLILQIGNII  SIWVSHSIQL  GNQSQIETCN  QSVITYENNT  WVNQTYVNIS  NTNFAAGQSV  VSVKLAGNSS
LCPVSGWAIY  SKDNSVRIGS  KGDVFVIREP  FISCSPLECR  TFFLTQGALL  NDKHSNGTIK  DRSPYRTLMS  CPIGEVPSPY  NSRFESVAWS
ASACHDGINW  LTIGISGPDS  GAVAVLKYNG  IITDTIKSWR  NNILRTQESE  CACVNGSCFT  IMTDGPSDGQ  ASYKIFRIEK  GKIVKSVEMN
APNYHYEECS  CYPDSSEITC  VCRDNWHGSN  RPWVSFNQNL  EYQIGYICSG  VFGDNPRPND  KTGSCGPVPS  NGANGVKGFS  FKYGNGVWIG
RTKSISSRKG  FEMIWDPNGW  TGTDNNFSIK  QDIVGINEWS  GYSGSFVQHP  ELTGLDCIRP  CFWVELIRGR  PEENTIWTSG  SSISFCGVNS
DTVGWSWPDG  AELPFTIDK

>OUTPUT_NA

MNPNQKIITI  GSVCMTIGMA  NLILQIGNII  SIWISHSIQL  GNQSQIETCN  QSVITYENNT  WVNQTYVNIS  NTNFAAGQSV  VSVKLAGNSS
LCPVSGWAIY  SKDNSVRIGS  KGDVFVIREP  FISCSPLECR  TFFLTQGALL  NDKHSNGTIK  DRSPYRTLMS  CPIGEVPSPY  NSRFESVAWS
ASACHDGINW  LTIGISGPDS  GAVAVLKYNG  IITDTIKSWR  NNILRTQESE  CACVNGSCFT  IMTDGPSDGQ  ASYKIFRIEK  GKIVKSVEMN
APNYHYEECS  CYPDSSEITC  VCRDNWHGSN  RPWVSFNQNL  EYQIGYICSG  IFGDNPRPND  KTGSCGPVSS  NGANGVKGFS  FKYGNGVWIG
RTKSISSRKG  FEMIWDPNGW  TGTDNNFSIK  QDIVGINEWS  GYSGSFVQHP  ELTGLDCIRP  CFWVELIRGR  PKENTIWTSG  SSISFCGVNS
DTVGWSWPDG  AELPFTIDK
```

(b)

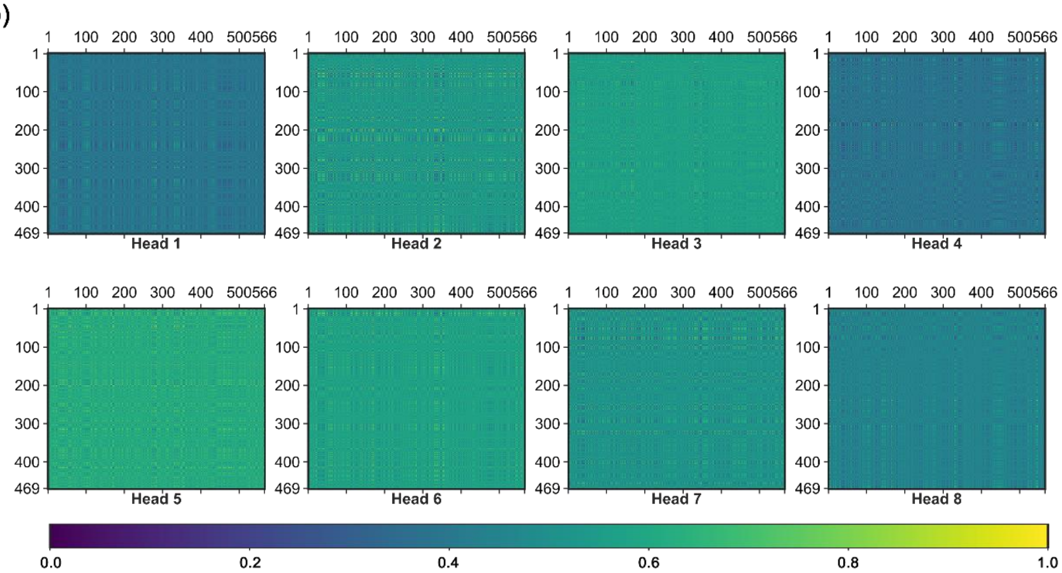

Figure S6. An example for "HA-to-NA" using sequence-to-sequence transformer model. (**a**) The HA protein of A/Wyoming/07/2013(H1N1) is selected as the input HA sequence ("INPUT_HA"), and its NA protein is targeted ("TARGET_NA"). The translation result is "OUTPUT_NA". Compared with "TARGET_NA", the different amino acids in "OUTPUT_NA" are marked red. (**b**) The multi-head attention weights. Each attention map is normalized to [0, 1].

(a)

```
>INPUT_NA

MNPNQKIITI  GSVCMTIGMA  NLILQIGNII  SIWVSHSIQL  GNQSQIETCN  QSVITYENNT  WVNQTYVNIS  NTNFAAGQSV  VSVKLAGNSS
LCPVSGWAIY  SKDNSVRIGS  KGDVFVIREP  FISCSPLECR  TFFLTQGALL  NDKHSNGTIK  DRSPYRTLMS  CPIGEVPSPY  NSRFESVAWS
ASACHDGINW  LTIGISGPDS  GAVAVLKYNG  IITDTIKSWR  NNILRTQESE  CACVNGSCFT  IMTDGPSDGQ  ASYKIFRIEK  GKIVKSVEMN
APNYHYEECS  CYPDSSEITC  VCRDNWHGSN  RPWVSFNQNL  EYQIGYICSG  VFGDNPRPND  KTGSCGPVPS  NGANGVKGFS  FKYGNGVWIG
RTKSISSRKG  FEMIWDPNGW  TGTDNNFSIK  QDIVGINEWS  GYSGSFVQHP  ELTGLDCIRP  CFWVELIRGR  PEENTIWTSG  SSISFCGVNS
DTVGWSWPDG  AELPFTIDK
```

```
>TARGET_HA

MKATLVVLLY  TFATANADTL  CIGYHANNST  DTVDTVLEKN  VTVTHSVNLL  EDKHNGKLCK  LRGVAPLHLG  KCNIAGWILG  NPECESLSTA
SSWSYIVETS  SSDNGTCYPG  DFINYEELRE  QLSSVSSFER  FEIFPKTSSW  PNHDSNKGVT  AACPHAGAKS  FYKNLIWLVK  KGNSYPKLSQ
SYINDKGKEV  LVLWGIHHPS  TTADQQSLYQ  NADAYVFVGT  SRYSKKFKPE  IAIRPKVRDQ  EGRMNYYWTL  VEPGDKITFE  ATGNLVVPRY
AFTMERNAGS  GIIISDTPVH  DCNTTCQTPE  GAINTSLPFQ  NIHPITIGKC  PKYVKSTKLR  LATGLRNVPS  IQSRGLFGAI  AGFIEGGWTG
MVDGWYGYHH  QNEQGSGYAA  DLKSTQNAID  KITNKVNSVI  EKMNTQFTAV  GKEFNHLEKR  IENLNKKVDD  GFLDIWTYNA  ELLVLLENER
TLDYHDSNVK  NLYEKVRNQL  KNNAKEIGNG  CFEFYHKCDN  TCMESVKNGT  YDYPKYSEEA  KLNREKIDGV  KLESTRIYQI  LAIYSTVASS
LVLVVSLGAI  SFWMCSNGSL  QCRICI
```

```
>OUTPUT_HA

MKAILVVLLY  TFATANADTL  CIGYHANNST  DTVDTVLEKN  VTVTHSVNLL  EDKHNGKLCK  LRGVAPLHLG  KCNIAGWILG  NPECESLSTA
SSWSYIVETS  SSDNGTCYPG  DFINYEELRE  QLSSVSSFER  FEIFPKTSSW  PNHDSNKGVT  AACPHAGAKS  FYKNLIWLVK  KGNSYPKLSQ
SYINDKGKEV  LVLWGIHHPS  TTADQQSLYQ  NADAYVFVGT  SRYSKKFKPE  IAIRPKVRDQ  EGRMNYYWTL  VEPGDKITFE  ATGNLVVPRY
AFTMERNAGS  GIIISDTPVH  DCNTTCQTPE  GAINTSLPFQ  NIHPITIGKC  PKYVKSTKLR  LATGLRNVPS  IQSRGLFGAI  AGFIEGGWTG
MVDGWYGYHH  QNEQGSGYAA  DLKSTQNAID  KITNKVNSVI  EKMNTQFTAV  GKEFNHLEKR  IENLNKKVDD  GFLDIWTYNA  ELLVLLENER
TLDYHDSNVK  NLYEKVRNQL  KNNAKEIGNG  CFEFYHKCDN  TCMESVKNGT  YDYPKYSEEA  KLNREKIDGV  KLESTRIYQI  LAIYSTVASS
LVLVVSLGAI  SFWMCSNGSL  QCRICI
```

(b)

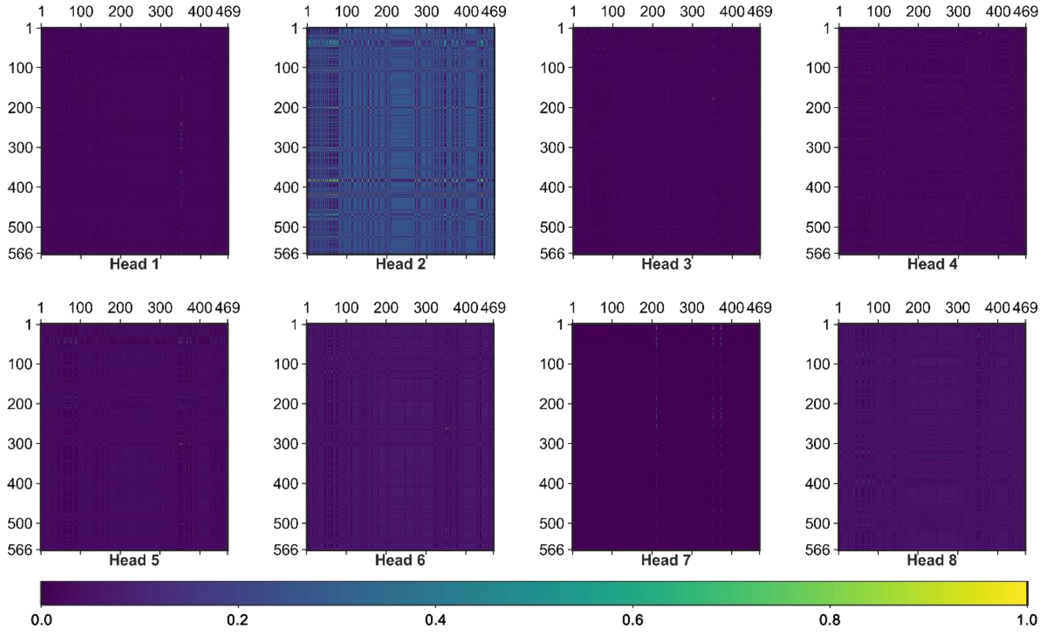

Figure S7. An example for "NA-to-HA" using sequence-to-sequence transformer model. (**a**) The NA protein of A/Wyoming/07/2013(H1N1) is selected as the input NA sequence ("INPUT_NA"), and its HA protein is targeted ("TARGET_HA"). The translation result is "OUTPUT_HA". Compared with "TARGET_HA", the different amino acids in "OUTPUT_HA" are marked red. (**b**) The multi-head attention weights. Each attention map is normalized to [0, 1].
